# Supplementary figures and images for: ybx1 acts upstream of atoh1a to promote the rapid regeneration of hair cells in zebrafish lateral-line neuromasts
Source: PeerJ. 2025 Sep 16;13:e19949. doi: 10.7717/peerj.19949 (PMC12447950; doi:10.7717/peerj.19949)

**Supplemental Tables**

**
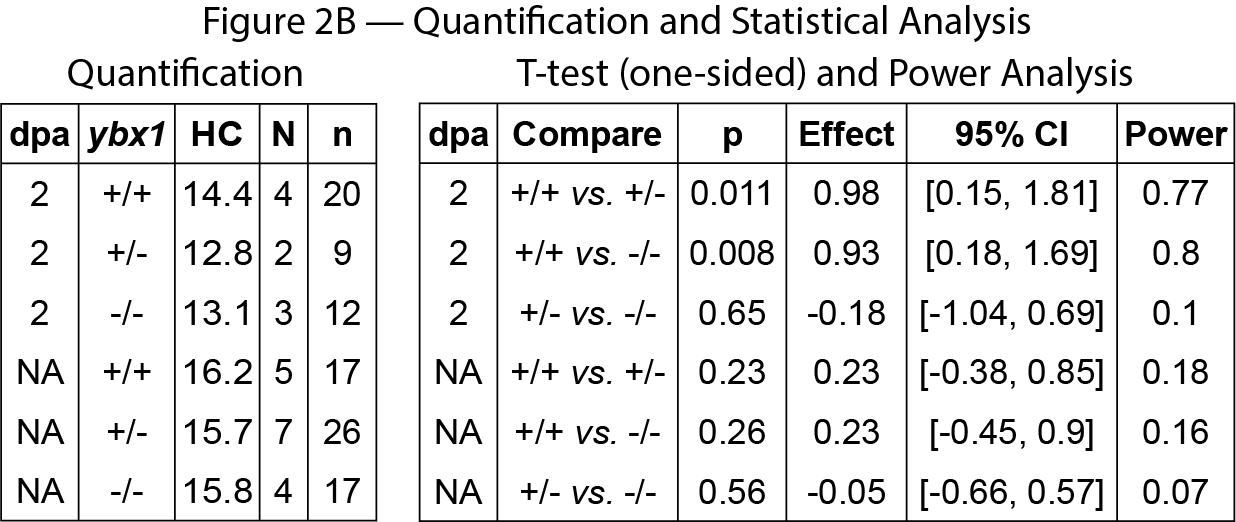
**

**
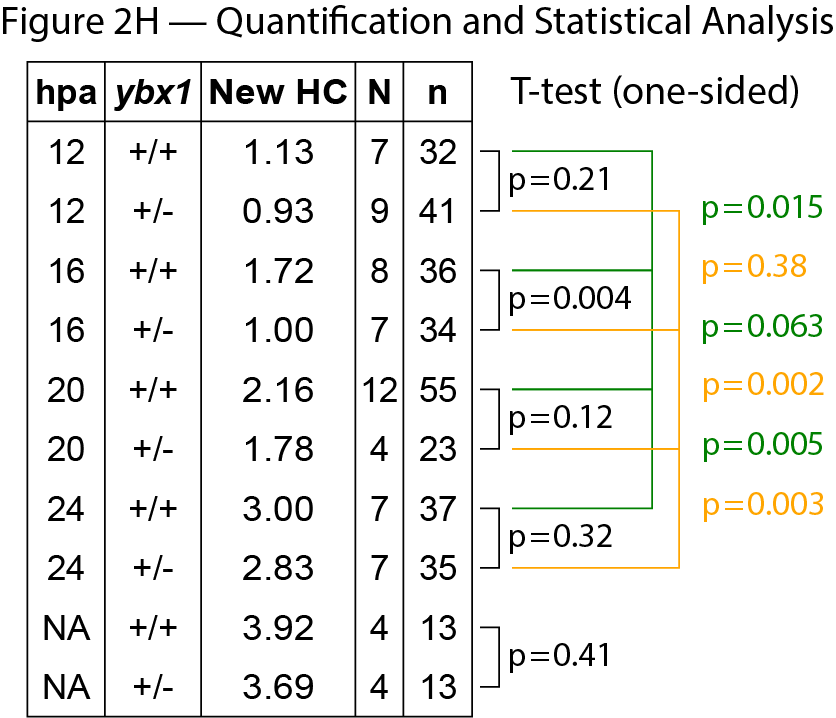
**

**
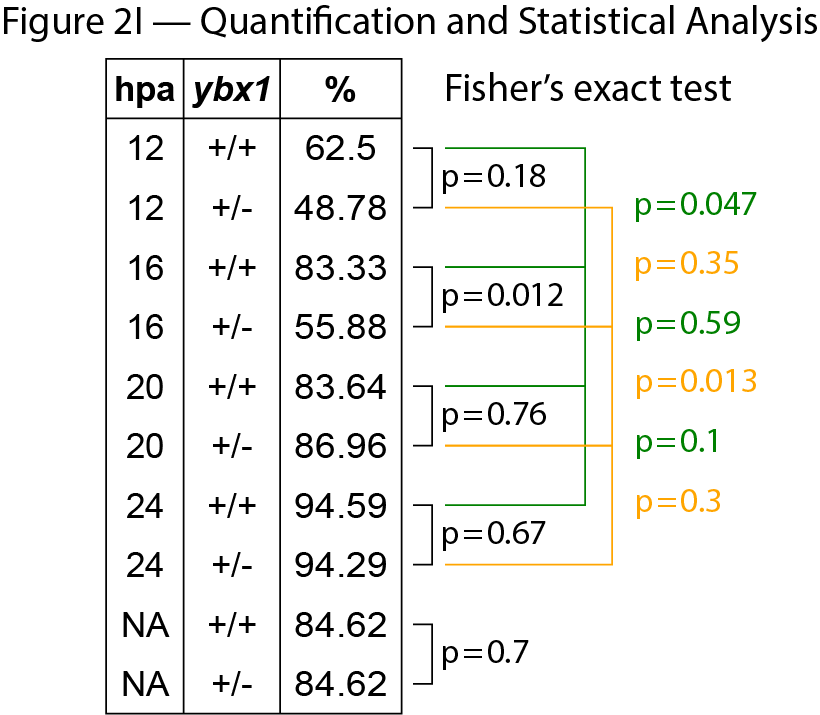
**

Supplement: Supplemental Information 1 [file peerj-13-19949-s001.docx]
